# Novel and Viable Acetylcholinesterase Target Site for Developing Effective and Environmentally Safe Insecticides

**DOI:** 10.2174/138945012799499703

**Published:** 2012-04

**Authors:** Yuan-Ping Pang, Stephen Brimijoin, David W Ragsdale, Kun Yan Zhu, Robert Suranyi

**Affiliations:** 1 Molecular Pharmacology and Experimental Therapeutics, Mayo Clinic, 200 First Street SW, Rochester, Minnesota, USA; 2 Department of Entomology, University of Minnesota, Saint Paul, Minnesota, USA; 3 Department of Entomology, Kansas State University, Manhattan, Kansas, USA; 4 McLaughlin Gormley King Company, Minneapolis, Minnesota, USA

**Keywords:** Pest control, disease vector control, anti-malaria, crop damage control, irreversible inhibitors, anticholinesterase.

## Abstract

Insect pests are responsible for human suffering and financial losses worldwide. New and environmentally safe insecticides are urgently needed to cope with these serious problems. Resistance to current insecticides has resulted in a resurgence of insect pests, and growing concerns about insecticide toxicity to humans discourage the use of insecticides for pest control. The small market for insecticides has hampered insecticide development; however, advances in genomics and structural genomics offer new opportunities to develop insecticides that are less dependent on the insecticide market. This review summarizes the literature data that support the hypothesis that an insect-specific cysteine residue located at the opening of the acetylcholinesterase active site is a promising target site for developing new insecticides with reduced off-target toxicity and low propensity for insect resistance. These data are used to discuss the differences between targeting the insect-specific cysteine residue and targeting the ubiquitous catalytic serine residue of acetylcholinesterase from the perspective of reducing off-target toxicity and insect resistance. Also discussed is the prospect of developing cysteine-targeting anticholinesterases as effective and environmentally safe insecticides for control of disease vectors, crop damage, and residential insect pests within the financial confines of the present insecticide market.

## 1 INTRODUCTION

New and environmentally safe insecticides are urgently needed worldwide to contend with the problems of disease-carrying, crop-destroying, and residential insect pests. For example, African malaria mosquitoes (*Anopheles gambiae* sensu stricto) transmit malaria, which sickens approximately 300 million and kills nearly 1 million people every year. Soybean aphids (*Aphis glycines*) cost U.S. farmers more than US$1 billion in yield losses and insecticide purchase and application. Bed bugs, which suck human blood and cause acute discomfort, mental stress, and social stigma, are now common in many residential areas. Insect resistance to current insecticides and growing concerns about insecticide toxicity to humans have contributed to a resurgence of insect pests.

Currently the cost of developing a traditional insecticide is approximately US$70 million, but the overall annual public health insecticide market for all diseases and all developing countries is just US$151 million [1]. This glimpse of the financial side of insecticide development and use suggests that a small return on investment is one of the key factors that hamper the development of new insecticides, especially those for public health purposes. Nevertheless, advancements in genomics and structural genomics offer new opportunities to identify targets at the structural level for insecticide development, a spin-off of medical genomics research that makes research and development incentives less dependent on the insecticide market. 

In this article, we review literature data pertaining to an insect-specific cysteine residue located at the rim of the acetylcholinesterase (AChE; EC 3.1.1.7) active site and the hypothesis that this residue is a novel target site for insecticide development. With these data, we discuss the differences between targeting the insect-specific cysteine residue and targeting the ubiquitous catalytic serine residue of AChE from the perspective of reducing off-target toxicity and insect resistance. We also discuss the prospect of developing cysteine-targeting anticholinesterases as effective and environmentally safe insecticides for controlling disease transmission, crop damage, and residential pests within the confines of the current insecticide market. 

## 2 INSECTS AS DISEASE VECTORS AND CROP AND RESIDENTIAL PESTS

### 2.1 Disease Vectors

Mosquitoes are disease vectors afflicting people in both developing and industrialized nations. For example, African malaria mosquitoes transmit malaria in sub-Saharan Africa [2], northern house mosquitoes (*Culex pipiens* L.) transmit St. Louis encephalitis [3] and West Nile virus [4] in North America, and yellow fever mosquitoes (*Aedes aegypti* L.) transmit dengue, yellow fever, and chikungunya [5] in most tropical and subtropical regions, including the United States. Recently, mosquito populations have surged owing both to the emergence of insect populations with resistance to current insecticides and to more and more restricted use of insecticides in response to concerns about environmental safety [6]. Novel insecticides are urgently needed to control mosquito-borne diseases, especially malaria, which contributed to the decline of the Roman empire and has caused grave concern in humans for 500,000 years [7]. According to the World Malaria Report 2010 [8], about 765 million of the world’s population is at risk of malaria, and an estimated 225 million cases led to nearly 781,000 deaths in 2009. 

### 2.2 Crop Pests

Aphids are insect pests of grain crops, vegetables, ornamental plants, and fruit trees. For 150 years the greenbug (*Schizaphis graminum*) has been a major pest of small grains (e.g., sorghum and wheat). Annual costs for greenbug control in wheat production are approximately US$100 million on the Texas High Plains alone [9]. The soybean aphid (*Aphis glycines*) costs more than US$1 billion in yield losses and insecticide expenditure in the United States [10]. During their reproduction season all aphids become female to produce progeny through asexual reproduction. This form of reproduction, which produces up to 18 asexual generations per growing season [11], allows aphids to develop insecticide resistance rapidly and demonstrates the inherent challenge in developing insecticides with low propensity for insect resistance. More advanced insecticides are needed to address this degree of resistance capability to ensure production of food and fiber for an ever-growing world population.

### 2.3 Residential Pests

Residential insect pests are of medical and economic importance. Wasps deliver painful stings and venom that can lead to allergenic responses and even death from anaphylactic shock [12]. *Tribolium* beetles infest flour and grain stores and contaminate food with carcinogenic quinoles [13-15]. Cockroaches deposit feces that become household allergens [16]. Regaining their formidable reputation as nearly ineradicable pests [17, 18], blood-feeding bed bugs (*Cimex lectularius*) can harbor up to 28 human pathogens and cause acute human discomfort from bites and feeding, delayed physical reactions to their saliva, allergen production, mental stress, and social stigma. Bed bug infestations have become increasing common in houses, apartments, hotels, hospitals, college dormitories, schoolrooms, and vehicles [19-21]. Current insecticides for controlling these residential pests are either ineffective or unsuitable for use close to food or inside dwellings because of the insecticide toxicity to humans or other mammals. The latter underscores the importance of reducing human and other off-target toxicities of new insecticides. 

## 3 INSECT-SPECIFIC CYSTEINE IN ACETYLCHOLINESTERASES

### 3.1 Acetylcholinesterase

**Fig. (1):**
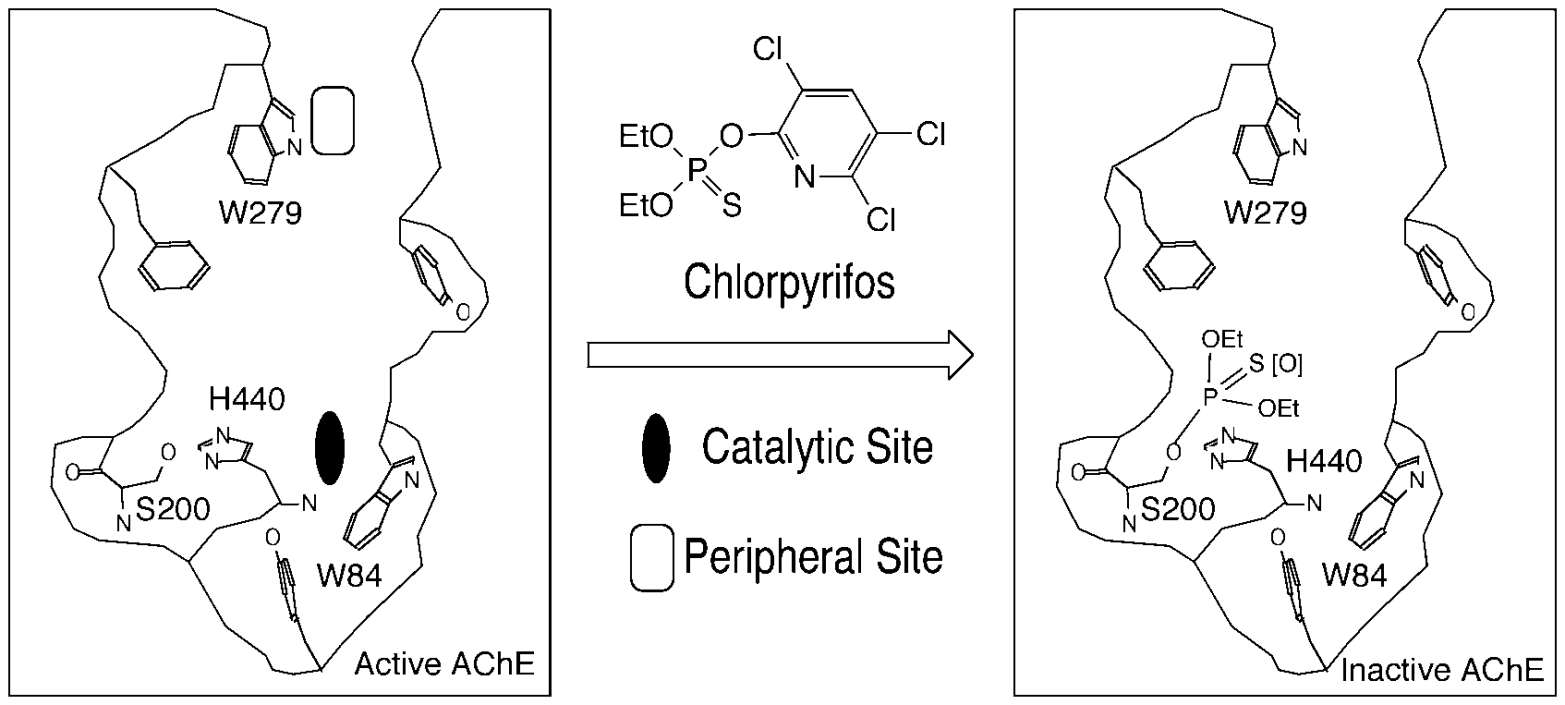
The active-site gorge of acetylcholinesterase showing the locations of the peripheral and catalytic sites and conjugation of chlorpyrifos with the catalytic serine reside.

AChE is a serine hydrolase vital for regulating the neurotransmitter acetylcholine in mammals, birds, and insects [22-24]. This enzyme has a deep and narrow active-site gorge (Fig. **1**), with a catalytic site at the bottom and a peripheral site at the entrance [22, 24, 25]. As a serendipitous outcome of World War II research on organophosphate nerve agents, current anticholinesterase insecticides—such as chlorpyrifos and methamidophos—work through phosphorylation of a serine residue at the AChE catalytic site (Fig. **1**), thus disabling the catalytic function and causing incapacitation. Because this serine residue is also ubiquitous in AChEs of mammals and other species with cholinergic nerves, the use of anticholinesterase insecticides to target the serine residue causes serious off-target toxicity. 

### 3.2 Two Insect Acetylcholinesterase Genes

Despite reports of resistance-conferring mutations in the only AChE gene of the fruit fly (*Drosophila melanogaster*) [26] and biochemical evidence of decreased AChE sensitivity to current insecticides in certain strains of the northern house mosquito [27, 28], no mutations were found in the AChE gene—which is orthologous to the fruit fly AChE gene—in these anticholinesterase-resistant strains [29]. The inability to identify resistance-conferring mutations in the orthologous gene eventually led to the “two-AChE-gene hypothesis” that resistance-conferring mutations occur in an unidentified gene paralogous to the fruit fly gene [30]. 

This hypothesis was based on the seminal discovery of the paralogous gene in the greenbug [31] and it was confirmed subsequently by the paralogous genes in the African malaria mosquito [32], the cotton aphid (*Aphis gossypii*) [33], the northern house mosquito [34], the green peach aphid (*Myzus persicae*) [35], the Japanese encephalitis mosquito (*Culex tritaeniorhynchus*) [36], the diamondback moth (*Plutella xylostella*) [37], the cotton bollworm (*Helicoverpa armigera*, AP-AChE: GenBank Accession No. AAY59530; AO: [38]), the codling moth (*Cydia pomonella*) [39], the German cockroach (*Blattella germanica*) [40], the human louse (*Pediculus humanus*) [29], the honeybee (*Apis mellifera*) [41], the oat aphid (*Rhopalosiphum padi*) [42], the English grain aphid (*Sitobion avenae*) [42], the yellow fever mosquito [43-45], the domestic silkworm (*Bombyx mori*) [46], the lesser mealworm (*Alphitobius diaperinus*) [47], the cat flea (*Ctenocephalides felis*) [48], the wild silkmoth (*Bombyx mandarina*) [49], the red flour beetle (*Tribolium castaneum*, AP-AChE: GenBank Accession No. XP_973462; AO-AChE: GenBank Accession No. XP_970774), the jewel wasp (*Nasonia vitripennis*, AP-AChE: GenBank Accession No. XP_001600458; AO-AChE: GenBank Accession No. XP_001605568), and the sand fly (*Lutzomyia longipalpis*) [50]. Further studies have suggested that the paralogous AChE gene is the predominant form of AChE, expressed in the greenbug [30], the diamondback moth [37], the human louse [29], and the insecticide-resistant Japanese encephalitis mosquito [51]. 

It is now well accepted that, unlike mammals and some flies, most disease-transmitting, crop-damaging, and residential insects have two AChE genes. The flies with one AChE gene include the fruit fly [52], the house fly (*Musca domestica*) [53], the horn fly (*Haematobia irritans*) [54], the olive fly (*Bactrocera oleae*) [55], the oriental fruit fly (*Bactrocera dorsalis*) [56], the Mediterranean fruit fly (*Ceratitis capitata*) [57], the stable fly (*Stomoxys calcitrans*, GenBank Accession No. ACT34084), and the Australian sheep blowfly (*Lucilia cuprina*) [58]. The labels ace-1 and ace-2 have been given to the paralogous genes in the literature; to avoid confusion, the insect AChE genes orthologous and paralogous to the AChE gene in the fruit fly are termed AO-AChE and AP-AChE, respectively, according to the nomenclature given by reference [29]. We use the AO and AP nomenclature hereafter.

### 3.3 Insect-Specific Cysteine Residue

A sequence alignment of nine AChE sequences (humans, the Florida lancelet [*Branchiostoma floridae*], the electric ray [*Torpedo californica*], the African malaria mosquito, the northern house mosquito, the greenbug, the cotton aphid, the green peach aphid, and the honeybee) identified a cysteine residue, Cys289 in the greenbug AP-AChE, that is absent in human AChE but conserved in insect and lancelet AChEs [59]. This alignment [59] along with site-directed mutagenesis work with lancelet AChE [59] and the previously reported sensitivity of aphid AChEs to sulfhydryl inhibitors [60, 61] collectively led to discussions on whether Cys289 or its equivalent in other aphid AP-AChEs is located in the active site [59] and whether it could be used as a target for developing selective aphidicides [62]. 

**Fig. (2):**
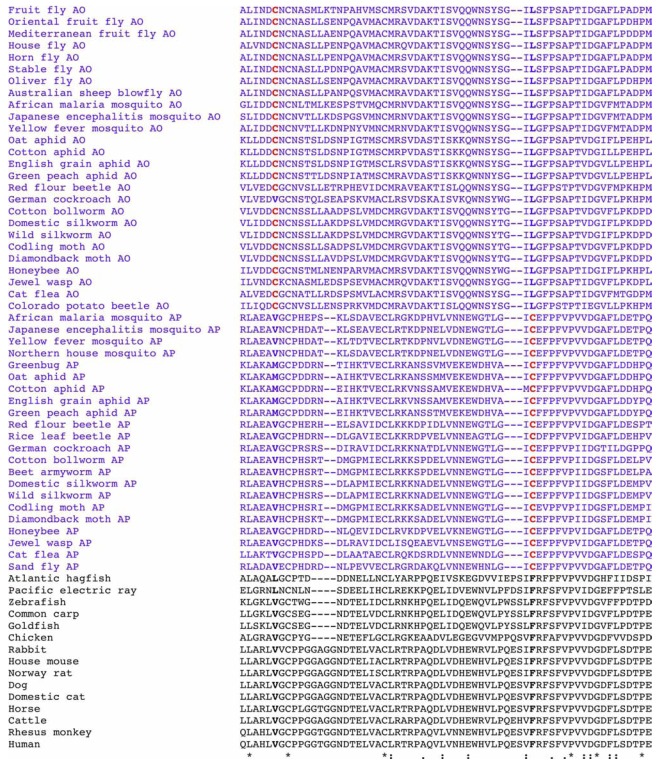
Multiple sequence alignment of acetylcholinesterases of insects and other species showing the sequence location of the insect-specific cysteine residue.

As apparent from the Protein Data Bank (PDB) deposition date of September 10, 2005 for the 3D mosquito AChE model (PDB ID: 2AZG) indicating that a large-scale sequence analysis and a subsequent 3D model prediction of mosquito AP-AChE were performed eight months before the online publication of reference [59] in 2006, an independent sequence analysis of 112 AChE sequences in different species identified the same cysteine residue that is absent in mammalian, fish, and avian AChEs but conserved in AP-AChEs of 16 insects–most of which are disease vectors as well as crop and residential pests (Fig. **2**) [63, 64]. The 16 insects with the conserved cysteine residue included the African malaria mosquito, the northern house mosquito, the Japanese encephalitis mosquito, the German cockroach, the rice leaf beetle (*Oulema oryzae*, AP-AChE: GenBank Accession No. AAN71602), the cotton bollworm, the beet army-worm (*Spodoptera exigua*, AP-AChE: GenBank Accession No. ABB86963), the codling moth, the diamondback moth, the domestic silkworm, the honeybee, the oat aphid, the greenbug, the cotton aphid, the green peach aphid, and the English grain aphid. Recent sequence analyses [48, 65] expanded the list to 22 insects with AP-AChEs that have a cysteine residue equivalent to Cys289 of the greenbug AP-AChE by adding the yellow fever mosquito, the cat flea, the wild silkmoth, the red flour beetle, the jewel wasp, and the sand fly. Although the AP-AChE sequences have not yet been reported, a recent biochemical study [66] provides direct evidence that yellow jacket wasp (*Vespula maculifrons*), Asian ladybird beetle (*Harmonia axyridis*), the American cockroach (*Periplaneta americana*), and the bed bug (*Cimex lectularius*) also have AP-AChE equivalent to that in the greenbug. 

Although hardly mentioned in the literature, a well-conserved cysteine residue that is 37 residues away in aligned sequence from Cys298 in the greenbug AP-AChE or its equivalent in other species is present in all AO-AChEs reported to date–including the AO-AChE in the Colorado potato beetle [67]–except for that in the German cockroach. According to our sequence alignment, which is shown for the first time in this review (Fig. **2**), this residue—Cys290 in the fruit fly AO-AChE—is mutated to a non-cysteine residue in AChEs of other species. Given the data reported to date, it is conceivable that most insect AChEs (AO or AP) have a well-conserved cysteine residue that is relatively close to (AO) or at (AP) the rim of the active site and that the corresponding residue in non-insect species is mutated to a hydrophobic residue. Hereafter we refer to the cysteine residue in AO-AChE or AP-AChE as the insect-specific cysteine residue. 

### 3.4 Crystal Structures and Computer Models of Insect Acetylcholinesterases

**Fig. (3):**
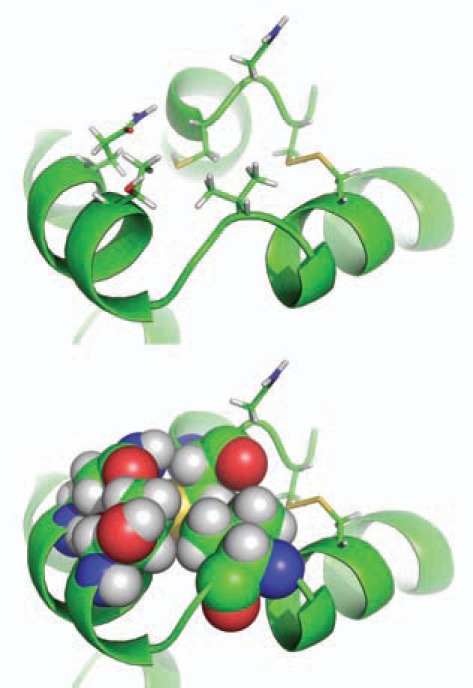
Close-up view of Cys290, Cys292, and Cys307 in the crystal structure of the fruit fly acetylcholinesterase (PDB ID: 1DX4) showing the physical blockage of Cys290 by Val311, Thr315 and Gln319.

While heterologous expressions of African malaria mosquito AP-AChE and some other insect AP-AChEs have been reported [48, 49, 68], the only three crystal structures of insect AChEs reported to date are the AO-AChE from the fruit fly in the free and bound states [69]. Interestingly, all three structures resolved at 2.7 Å show that Cys290 in the fruit fly AO-AChE—the insect-specific cysteine residue—is completely buried by Val311, Thr315, and Gln319 (Fig. **3**), while its neighbor, Cys292, forms a disulfide bond with Cys307. In addition, Cys290 remained the buried conformation in all the trajectories saved at 1.0-ps intervals of three independent 2-ns-long simulations of the *apo* fruit fly AO-AChE crystal structure [Protein Data Bank (PDB) ID: 1QO9 [69], Yuan-Ping Pang’s unpublished work]. These observations indicate that the insect-specific cysteine residue in fruit fly AO-AChE structures is inaccessible to sulfhydryl agents. Considering Cys289 in the greenbug AP-AChE or its equivalent in other AP-AChEs as an insecticide target sites would be inappropriate without a crystal structure or a credible computer model of an AP-AChE to ensure that the insect-specific cysteine residue is not blocked from conjugation with sulfhydryl agents by neighboring residues or bonded to a spatially nearby cysteine.

Homology modeling and effective multiple molecular dynamics simulation refinement led to a set of computer models of AP-AChEs that were made freely available at the PDB and published together with the large-scale sequence analysis described in Section 3.3 [63, 64]. This set includes greenbug (PDB ID: 2HCP), English grain aphid (PDB ID: 2HCQ), and African malaria mosquito (PDB ID: 2AZG) AP-AChEs. 

**Fig. (4):**
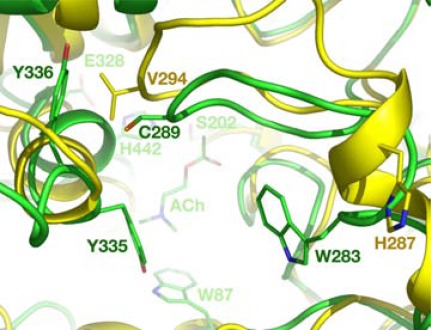
Overlay of the greenbug (green) and human (yellow) acetylcholinesterases from a perspective looking down onto substrate acetylcholine at the catalytic site.

In the models of greenbug and English grain aphid AP-AChEs, the insect-specific cysteine residue—Cys289 in greenbug AP-AChE—is located at the entrance of the AP-AChE active site [64]. In the human AChE crystal structure [70], the residue spatially corresponding to Cys289 is Val294 (Fig. **4**). Most importantly, unlike Cys290 in fruit fly AO-AChE, Cys289 in these models is not completely buried by neighboring residues, and it is spatially remote to any cysteine residues for a disulfide bond formation. In other words, Cys289 is accessible to sulfhydryl agents for conjugation. 

**Fig. (5):**
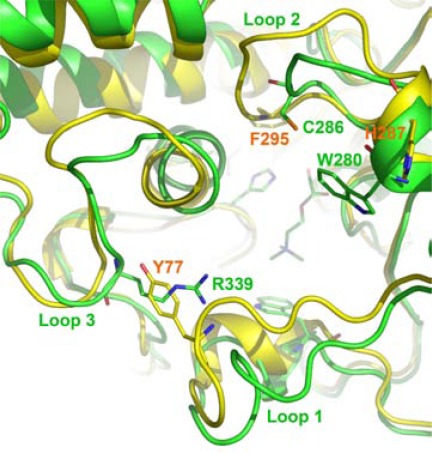
Overlay of the African malaria mosquito (green) and human (yellow) acetylcholinesterases from a perspective looking down onto substrate acetylcholine at the catalytic site.

Similarly, the model of African malaria mosquito AP-AChE shows that its insect-specific cysteine residue is unpaired and accessible to electrophiles binding at the active site (Fig. **5**) [63]. The spatial equivalent of Cys286 in human AChE is Val294 or Phe295 (Fig. **5**). These mosquitoes also have an arginine residue (Arg339 of malaria mosquito AP-AChE) at the rim of the AP-AChE active site that appears to be genus specific (see Section 6.3 for discussion on the implication of Arg339) [63].

**Fig. (6):**
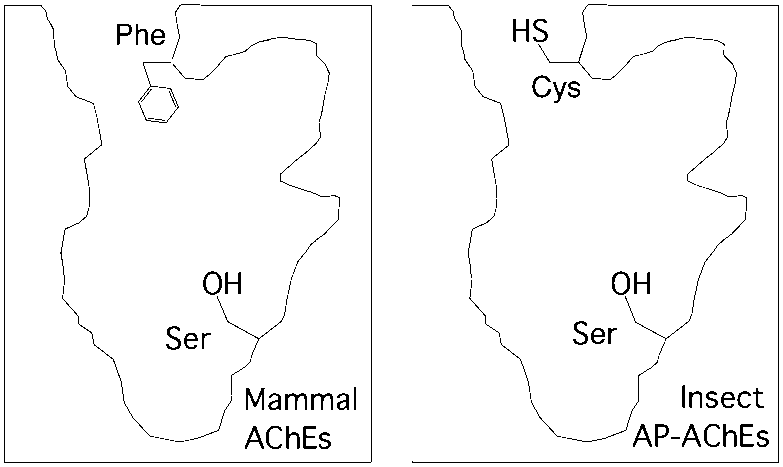
Structural difference at the entrance of the active site between mammalian and insect acetylcholinesterases.

By extrapolating the results above, it is conceivable that insect AP-AChEs have a cysteine residue “guard” at the entrance of the active site, whereas mammalian AChEs have a phenylalanine residue as an “usher” for cationic ligands at the entrance (Fig. **6**). This structural difference between mammal and insect species offers a new opportunity for developing effective and environmentally safe insecticides as described below.

## 4 INSECT-SPECIFIC CYSTEINE AS A NEW TARGET SITE

It was known that a native or engineered cysteine residue near or at the active site of an enzyme can covalently bond to a small molecule that binds, even loosely, at the active site, as long as that molecule carries a sulfhydryl moiety [71] or a leaving group that is vulnerable to attack by a thiol group [72]. Sulfhydryl reagents are also known to form adducts with a cysteine residue at the peripheral site of a mammalian AChE engineered with a His287Cys mutation and thereby to interfere with substrate binding and consequent catalytic activity [73, 74]. 

In this context, the hypothesis that the insect-specific cysteine residue located at the opening of the AP-AChE active site would be a promising target site for developing new insecticides was reported and discussed [63, 64]. Initial support for this hypothesis came from sequence analysis [63, 64], computer-generated 3D models of AP-AChEs [63, 64], and demonstrations of sulfhydryl agents inhibiting enzyme catalysis by conjugation with a native or engineered cysteine residue at the active site [71, 72] and of AChE inhibition through the blockage of the AChE peripheral site [73, 74]. 

The advantage of this hypothesis is at least fourfold. First, insecticides that target the insect-specific cysteine residue should be less toxic to mammals than current anti-cholinesterases that target the ubiquitous catalytic serine residue of all AChEs. Second, targeting the cysteine residue alleviates the resistance problems that have occurred with current serine-targeting insecticides that have been used for decades, because insects have had no opportunity to develop resistance to cysteine-targeting insecticides. Third, most insects appear to have AP-AChE with a free cysteine residue at the entrance of the active site; this enables a generic approach to developing cysteine-targeting insecticides. Finally, the hypothesis offers a low-cost of development of new insecticides. Given the small size of the insecticide market, this advantage is the most important, as adequate financial resources are currently unavailable for the development of individualized approaches to a wide array of problems caused by insects. 

## 5 INHIBITION OF INSECT ACETYLCHOLINESTERASES

### 5.1 Irreversible Inhibition of Insect Acetylcholinesterase Activity

**Fig. (7):**
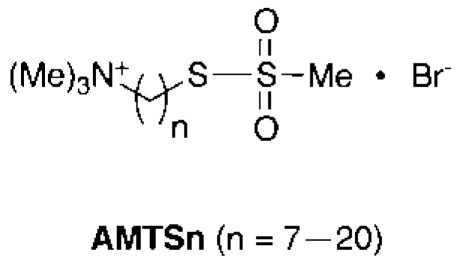
Chemical structures of **AMTS7**–**AMTS20**.

To test the hypothesis that the insect-specific cysteine residue is a viable target site, prototypic irreversible inhibitors (**AMTS7**–**AMTS20; **Fig. **7**) were reportedly made to investigate whether (1) Cys289 in greenbug AP-AChE is indeed accessible for conjugation, (2) inhibition of AP-AChE can deplete greenbug AChE activity entirely, and (3) these prototypic cysteine-targeting inhibitors can affect human AChE activity [65]. Inspired by the work of reference [74], **AMTS7**–**AMTS20** were designed to have a trimethyl-ammonium group to confer affinity by the cation-pi interaction with Trp87 at the active site of greenbug AP-AChE [22, 24] and a methanethiosulfonate group known to form an adduct preferentially with free cysteine residues [75]. A crystal structure of **AMTS13** in complex with recombinant mouse AChE resolved at 2.6 Å [76] suggests that these compounds have the ability to anchor the ammonium group atop the indole ring of Trp87 and place the methanethiosulfonate group in the vicinity of the free cysteine residue at the peripheral site. 

These inhibitors were reportedly tested for irreversible AChE inhibition by exposure to the total AChE-containing homogenate of insect or mammalian samples for a defined period of time, after which the unbound inhibitor was removed from AChE via extended dialysis [65]. A radiometric method devoid of artifacts caused by free thiol groups in samples or reagents was used to determine total and irreversible AChE inhibition in assays of AChE activity in the preparations before and after dialysis, respectively [65]. 

**Fig. (8):**
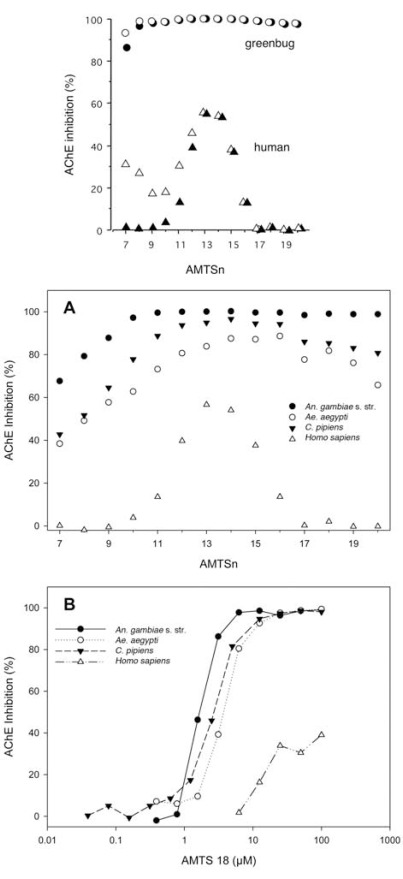
Effects of inhibitor length and concentration on AChE inhibition by **AMTSn**. **A:** the length effect on the greenbug and human AChE inhibition; **B:** the length effect on the mosquito and human AChE inhibition; **C:** the concentration effect on the mosquito and human AChE inhibition.

As shown in Fig. (**8A**), the long-chain inhibitors (**AMTS17**-**AMTS20**) at 6 µM achieve nearly total and irreversible inhibition of greenbug AChE but hardly affect human AChE under identical conditions. These inhibitors also show 95% inhibition of the total AChE activity in the African malaria mosquito and >80% inhibition in northern house and the yellow fever mosquitoes (Fig. **8B**). In addition, the long-chain inhibitors exhibit selective irreversible inhibition of total AChE activity in soybean aphids at the same inhibitor concentration [65]. 

Unexpectedly, the short- and medium-chain inhibitors (**AMTS7**–**AMTS16**) show slow, partial, and irreversible inhibition of human AChE as well. Although **AMTS17**–**AMTS20 **at 6 µM showed no irreversible inhibition of human AChE [65], a full dose-response analysis detected irreversible inhibition at inhibitor concentrations of >10 µM (Fig. **8C**). The irreversible inhibitory effect on the human enzyme by **AMTS17**–**AMTS20** at 100 µM is similar to that by **AMTS13** at 6 µM [76]. 

**Fig. (9):**
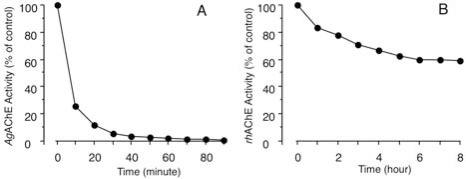
Time courses of irreversible inhibition for mosquito and human AChEs by **AMTS13**. Extracts of African malaria mosquito AChE (*Ag*AChE) and recombinant human AChE (*rh*AChE) were exposed, respectively, to 6 µM **AMTS13** at room temperature for defined intervals of time terminated by addition of glutathione (to a final concentration of 500 µM). Shown is AChE activity in treated samples assayed radiometrically.

Furthermore, **AMTS13** reportedly conjugates with glutathione within seconds, although by itself glutathione has no detectable effect on AChE activity in extracts from mosquitoes, human red blood cells, or recombinant human AChE [76]. This observation led to determination of the time course of irreversible AChE inhibition by **AMTS13**. Of mechanistic importance, 6 µM **AMTS13** causes rapid and irreversible inhibition of African malaria mosquito AChE, reaching ~50 and ~100% irreversible inhibition within 10 and 30 minutes, respectively (Fig. **9A**). By contrast, a four-hour incubation was needed to achieve 30% irreversible inhibition of recombinant human AChE (Fig. **9B**). Thus, irreversible inhibition of mosquito AChE by 6 µM **AMTS13** is fast and nearly complete, whereas that of human AChE is slow and partial. 

Given the complication of an unexpected irreversible inhibition of the human enzyme, reactivation experiments were necessarily performed using greenbug AChE samples, **AMTS13, **and 2-mercaptoethanol [75], a disulfide bond reducing (breaking) agent, to determine whether the observed irreversible AChE inhibition was due to the expected conjugation to the insect-specific cysteine residue [65]. The reported data shown in Table **1** [65], demonstrate that when fresh greenbug and red blood cell extracts were treated for two hours with 100 mM 2-mercaptoethanol and then dialyzed, the greenbug AChE pretreated with **AMTS13** was dramatically reactivated by 2-mercaptoethanol, whereas the human AChE activity fell to 21% of the control. The effect of 2-mercaptoethanol on the human enzyme is understandably caused by the rupture of the solvent-exposing disulfide bond in AChE by 2-mercaptoethanol that consequently reduces the AChE activity, although 2-mercaptoethanol can at the same time cleave the **AMTS13** conjugate to reverse the inhibition of greenbug AChE by **AMTS13** [76]. A similar result was shown for a mosquito AChE sample [76]. 

Collectively, the results of the reactivation studies using aphid and mosquito samples demonstrate that the inhibition of greenbug or mosquito AChEs by **AMTS13** resulted from conjugation of the [(Me)_3_N^+^(CH_2_)_13_S–] fragment of **AMTS13** to the insect-specific cysteine residue of AP-AChE. The time course and reactivation studies show that the slow, partial, and irreversible inhibition of human AChE by short- and medium-chain **AMTS7**–**AMTS16 **at low concentrations or by long-chain **AMTS17**–**AMTS20** at high concentrations is caused by different inhibition mechanisms. 

### 5.2 Mechanisms of Irreversible Inhibition

Although the reported slow, partial, and irreversible inhibition of human AChE by **AMTS13** appeared oracular and initially led to incorrect speculation about a reaction of **AMTS13** with the catalytic serine residue of AChE [65], further studies using 2-mercaptoethanol, atomic force microscopy, circular dichroism spectroscopy, X-ray crystallography, time-resolved fluorescence spectroscopy, and liquid chromatography triple quadrupole mass spectrometry reportedly identified two distinct mechanisms for the observed irreversible inhibition of AChEs [76]. 

The fast, nearly complete, and irreversible inhibition of African malaria mosquito AChE by 6 µM **AMTS13** is initiated by a reversible interaction between the ammonium group of **AMTS13** and Trp84 placing the methanethiosulfonate group at the rim of the active site. This positioning puts the reactive group close to the insect-specific cysteine residue and facilitates a rapid conjugation of the inhibitor with the mosquito AP-AChE. This disulfide bond formation mechanism is primarily supported by the reactivation of the **AMTS13**-inhibited insect AChE by 2-mercaptoethanol and data from the crystal structure of **AMTS13 **in complex with mouse AChE.

The slow, partial, and irreversible inhibition of human AChE by 6 µM **AMTS13** is caused by partial denaturation of the enzyme induced by **AMTS13** and/or micelles of **AMTS13**. Evidence of this mechanism comes primarily from far-UV circular dichroism spectra that show a reduction of secondary structures in recombinant mouse AChE treated with **AMTS13** for 45 hours as well as from atomic force microscopy results indicating that **AMTS13** has a high propensity for micelle formation. This tendency is consistent with the report that alkyltrimethylammonium halides with 12–16 methylenes—in particular dodecyl-trimethylammonium halides—are effective surfactants that can denature α-lactalbumin or β-lactoglobulin [77, 78]. Although methane-thiosulfonate is slightly hydrophilic, the overall structure of **AMTS13** is analogous to that of dodecyl-trimethyl-ammonium halides. 

## 6 DEVELOPMENT OF CYSTEINE-TARGETING INSECTICIDES

### 6.1 Support for the Cysteine-Targeting Hypothesis

Despite the complication of surfactant-like inhibitors causing slow, partial, and irreversible inhibition of human AChE and a report that iodoacetamide-containing AChE inhibitors do not bond covalently to African malaria mosquito AP-AChE [79], the fast, nearly full, and irreversible inhibition of the insect AChE activity by **AMTSn** together with the reactivation of **AMTSn**-inhibited AChEs by 2-mercaptoethanol unequivocally show that the insect-specific cysteine residue is, as shown in independent 3D models of the AP-AChEs, indeed *accessible* for conjugation with sulfhydryl agents [65, 76]. In theory cysteine-targeting insecticides can be developed to minimize off-target toxicity by taking advantage of the species difference in AChEs. 

At this point, it is fair to question whether selectively inhibiting AP-AChE but not AO-AChE can deplete total insect AChE activity and lead to insect incapacitation. Consistent with the reported preponderance of AP-AChE over AO-AChE [29, 30, 37, 51], **AMTS18** irreversibly inactivated >80% of the total AChE activity in greenbug and mosquito extracts [65, 76]. This observation could be due to either that AO-AChE is poorly extracted and not measured in the assay or that AO-AChE is a minor contributor to the total acetylcholine-hydrolysis activity in insects. The former appears unlikely for several reasons. The extraction conditions used extensive mechanical homogenization to create fine suspensions from greenbug and mosquito samples in which all of the AChE should have been accessible to the substrate. The assays were performed directly on the suspensions without first removing insoluble matter using centrifugation or filtration. In the reported preliminary experiments with the fruit fly—the well-characterized genome of which includes *only* AO-AChE [52]—the identical extraction protocol rendered abundant fruit fly AChE activity that was resistant to **AMTS18**. It is therefore conceivable from the reported data that inhibition of AP-AChE can deplete total insect AChE activity in greenbug and mosquitoes. This means that selectively inhibiting AP-AChE is at least theoretically adequate to incapacitate insects with cholinergic nerves.

To support the cysteine-targeting hypothesis further, it is necessary to address the reported reservations about targeting the insect-specific cysteine residue [62]. If the understanding of reference [62] is correct, the concerns are about (1) the poor reaction rates of two known sulfhydryl agents, 5,5′-dithiobis(2-nitrobenzoic acid) and *N*-ethylmaleimide, for conjugation with Florida lancelet AP-AChE and (2) an active-site phenylalanine residue that hampers the reactions of these two reagents with the insect-specific cysteine residue. Neither 5,5′-dithiobis(2-nitrobenzoic acid) nor *N*-ethylmaleimide is designed for insect AP-AChEs, and their reaction rates with the lancelet enzyme and the steric hindrance experienced in the lancelet enzyme appear to be irrelevant to cysteine-targeting insecticides. What is relevant is the reaction rates of sulfhydryl agents tailored for insect AP-AChEs. As shown in Fig. (**9A**), 6 µM **AMTS13** causes rapid irreversible inhibition of African malaria mosquito AChE achieving 50% irreversible inhibition within 10 minutes. This fast conjugation with insect enzymes demonstrated by a primitive cysteine-targeting inhibitor indicates that sufficient rates for the reaction of sulfhydryl agents with the insect-specific cysteine residue should not be a theoretical or practical concern.

### 6.2 Targeting Cysteine *versus* Serine Residues of Acetylcholinesterase 

Mechanistically, targeting cysteine and serine residues uses the same chemical approach—irreversibly modifying an active-site residue leading to physical blockage of the active site and subsequent inactivation of the enzyme catalysis. In addition, the two approaches share the same enzyme. These commonalities can lead to the incorrect perception that targeting the cysteine residue of AP-AChE is the same as targeting the serine residue of AChE and that cysteine-targeting insecticides are as same as current anticholinesterase insecticides. It is worthy of noting the fundamental differences between these two approaches, however. 

First, cysteine is chemically more reactive than serine. This increased reactivity means that the cysteine-targeting chemicals can be made less reactive and should, therefore, have less off-target toxicity than serine-targeting chemicals such as organophosphates or carbamates. This disparity in chemical reactivity also means that cysteine-targeting chemicals are structurally different from those that target the serine residue, which magnifies their difference from current serine-targeting insecticides because cysteine-targeting insecticides will, at least initially, face no insect resistance conferred by the established metabolic detoxification.

Second, the locations of the two residues in the AChE active site are different, meaning that resistance to current serine-targeting insecticides conferred by mutations in the active site of AChE—for example, the Gly119Ser mutant of African malaria mosquito AP-AChE (GenBank ID: AJ515149 [34])—will not affect cysteine-targeting inhibitors. According to reported models of insect AP-AChEs [63, 64], the serine residue is located at the bottom of the active-site gorge, whereas the cysteine residue is situated at the opening of the gorge, and the separation between the alpha carbons of the two residues ranges from 15 to 18 Å. Structurally, mutating the active-site residues to prevent serine-targeting chemicals from entering the deep gorge is easier than to impeding agents from reaching the peripheral cysteine. It is fair to argue that insects could respond to the selective pressure of a cysteine-targeting agent by increased expression of AO-AChE. This response appears unlikely, however, as the northern house mosquito has developed its resistance to current insecticides through mutations of AP-AChE not by elevating levels of AO-AChE [27-29]. It is therefore conceivable that cysteine-targeting chemicals have a lower propensity for insect resistance than serine-targeting chemicals.

Despite some commonalities with serine-targeting insecticides, cysteine-targeting insecticides have lower off-target toxicity and decreased likelihood for insect resistance than serine-targeting insecticides owing to their relative chemical reactivity and the location of the insect-specific cysteine residue.

### 6.3 The Prospect of Developing Generic Cysteine-Targeting Insecticides

Given the discovery process of the insect-specific cysteine residue and its prospects as a new target for insecticide development, it may be tempting to use genomics and structural genomics to discover insecticide targets other than AChE. One should not overlook at the current fact that limited financial resources are available for insecticide development, however. Although new insecticide targets can be discovered using genomic and structural genomic studies that are now low-cost and “off-the-shelf” commodities, the expenses involved in developing a new chemical entity as an effective and environmentally safe insecticide can be formidably high and potentially impractical. Conversely, AChE is a well-studied enzyme and abundant literature details the development of effective AChE inhibitors, some of which are used currently as insecticides or others as clinical drugs for treating Alzheimer’s disease. Developing insecticides that target a different residue in AChE should be less costly than developing chemicals that target proteins that are not as well studied as AChE. For the financial and scientific reasons described in Sections 2–5, we advocate the pursuit of generic cysteine-targeting insecticides for effective and broad control of disease vectors and crop and residential insect pests.

Presently no reports on cysteine-targeting chemicals that effectively incapacitate insects without measurable mammalian toxicity have been published, partly because much of the current research effort is still directed toward proof of concept. Additionally, unlike current anticholinesterase insecticides that are low-hanging fruits stemming from World War II research on nerve gas, cysteine-targeting chemicals are high-hanging fruits for the following reasons. First, most known alkylating agents or electrophiles cannot be used to target the cysteine residue because these agents, such as Michael acceptors [80], can cause significant human toxicity. Second, it is disadvantageous to establish affinity by designing a long-chain molecule such as bis-(7)-tacrine [25] that binds deep in the active-site gorge, because such molecules are associated with a high likelihood that insects will develop resistance through active-site mutations of AChE, as mentioned above. Third, cysteine-targeting agents must have the capability to react quickly with cysteine, which opposes the low-reactivity requirement of low off-target toxicity. A delicate balance exists among these three factors in designing cysteine-targeting insecticides. Nevertheless, given recent advancements in structure-based molecular design such as synthesis-based computer-aided molecular design that accounts the feasibility and efficiency of inhibitor synthesis in addition to inhibitor affinity and selectivity [81], we think that cysteine-targeting insecticides with in vivo efficacy will be forthcoming. 

It has been recognized that honeybees and silkworms also have AP-AChEs with the insect-specific cysteine residue [63]. This fact raises a legitimate concern that cysteine-targeting insecticides could harm beneficial insects. It has been reported that mosquitoes have an arginine residue at the rim of the AP-AChE active site that appears to be genus-specific [63]. It has also been proposed that AP-AChE inhibitors can be designed to target two mosquito-specific residues (e.g., Cys286 and Arg339 in African malaria mosquito AP-AChE) or target an insect-specific residue (Cys289 in greenbug AP-AChE) [76] and an aphid-specific conformation of an active-site residue in the greenbug enzyme [65] to reduce toxicity to non-target insects. The essence of these approaches is the creation of individualized insecticides that hold great promise for controlling insects without off-target toxicity and insect resistance. After systemic considerations, however, we think it is currently impractical to pursue an individualized insecticide paradigm owing to the financial restrictions described in Section 1. 

Fortunately, honeybees and silkworms do not share habitat with mosquitoes or residential pests. A generic cysteine-targeting insecticide has to be developed first, and the deployment of this type of insecticide must be carefully managed to avoid toxicity to honeybees, silkworms, and other beneficial insects. A recent study shows that the observed differences in inhibitory potency by the prototypic irreversible inhibitor, **ATMSn**, do not exceed 8-fold [66]. This relatively narrow range suggests that the feasibility of developing a generic irreversible inhibitor that targets the insect-specific cysteine residue of AP-AChE in various insect pests is feasible. Hopefully, recognition of a new generation of insecticides with low off-target toxicity and low propensity for insect resistance will grow the insecticide market to the extent that individualized insecticides can eventually be pursued to reduce toxicity to beneficial insects.

## 7 CONCLUSION

Unlike mammals, most disease-transmitting, crop-damaging, and residential insects have AO- and AP-AChEs, each of which has an insect-specific cysteine residue. The cysteine of AP-AChE, located at the entrance to the active site, is accessible for rapid conjugation with sulfhydryl agents and represents a new opportunity to develop cysteine-targeting insecticides. Because cysteine is more reactive than serine—with which current anticholinesterase insecticides are designed to react—cysteine-targeting insecticides have lower off-target toxicity and a lower propensity for insect resistance than serine-targeting insecticides. Although the development of cysteine-targeting insecticides is not easy and the small insecticide market places financial constraints on research and development, the abundant literature on AChE inhibitor design and the cost-effectiveness of the development process make the insect-specific cysteine residue a viable target for developing effective and environmentally safe insecticides to control disease vectors, crop damage, and residential insect pests. 

## NOTE

The cysteine-containing AP-AChE list now includes those of **Asian tiger mosquito** (*Aedes albopictus*, GenBank Accession Number [GBAN]: AB218421), **bed bug **(*Cimex lectularius*, GBAN: AEN69455, JQ349158 [Harlan strain], and JQ349159 [Harlan strain]), booklice (*Liposcelis bostrychophila*, GBAN: FJ647185;* Liposcelis decolor*, GBAN: FJ647186; *Liposcelis entomophila*, GBAN: EU854149), **human body louse** (*Pediculus humanus corporis*, GBAN: AB266606), **human head louse **(*Pediculus humanus capitis*, GBAN: AB266615), oriental tobacco budworm (*Helicoverpa assulta*, GBAN: DQ001323), **southern house mosquito** (*Culex quinquefasciatus*, GBAN: XM_001847396), springtail (*Orchesella villosa,* GBAN: ACL27226), **soybean aphid** (*Aphis glycines*, GBAN: JQ349160), striped riceborer (*Chilo suppressalis*, GBAN: EF453724), and whitefly (*Bemisia tabaci*, GBAN: ABV45413).

## Figures and Tables

**Table 1.** Reactivation Studies of Greenbug and Human AChEs Inhibited by AMTS13 Using 2-Mercaptoethanol (BME)

Sample	Pretreatment	Treatment	% AChE activity
Greenbug	None	None	100
	“	BME 0.5 hr	36
	“	BME 1 hr	31
	“	BME 2 hr	21
	**AMTS13**	None	1
	“	BME 0.5 hr	12
	“	BME 1 hr	17
	“	BME 2 hr	16
Human	None	None	100
	“	BME 0.5 hr	49
	“	BME 1 hr	44
	“	BME 2 hr	35
	**AMTS13**	None	57
	“	BME 0.5 hr	28
	“	BME 1 hr	26
	“	BME 2 hr	20

The greenbug and human red blood cell extracts were exposed to **AMTS13** (6.0 µM) for 1 hr and/or to BME (100.0 mM) for different periods of time. After the exposure(s), samples were dialyzed overnight and the AChE activity was measured. Activities are mean values of triplicate determinations expressed as percentages of the AChE activity.
